# Synthetic Small-Molecule Ligands Targeted to Adenosine Receptors: Is There Potential Towards Ischemic Heart Disease?

**DOI:** 10.3390/cells14151219

**Published:** 2025-08-07

**Authors:** Qi Xu, Yaw Nana Opoku, Kalwant S. Authi, Agostino Cilibrizzi

**Affiliations:** 1 Institute of Pharmaceutical Science, King’s College London, Stamford Street, London SE1 9NH, UK; q.xu@kcl.ac.uk (Q.X.); yaw.opoku@kcl.ac.uk (Y.N.O.); 2 BHF Centre for Research Excellence, School of Cardiovascular and Metabolic Medicine and Sciences, King’s College London, Stamford Street, London SE1 9NH, UK; kalwant.authi@kcl.ac.uk

**Keywords:** adenosine, adenosine receptors, small-molecule ligands, adenosine targeting, synthetic ligands, ischemic heart disease, adenosine signalling

## Abstract

Ischemic heart disease (IHD) represents a leading cause of global morbidity and mortality. Despite significant advances in treatment achieved over recent decades, as well as various therapeutic strategies available to manage IHD progression currently, the global incidence of this disorder remains high. This review examines essential cell biology aspects of adenosine receptors (ARs), along with the effects of known synthetic small-molecule AR ligands, to provide an up-to-date view on the therapeutic potential towards IHD treatment. In particular, we report here advancements made on a selection of AR synthetic ligands that have demonstrated efficacy in pre-clinical or clinical studies, thereby holding promise as new therapeutic candidates in the field of IHD. Although this work adds further evidence that clinically valid small-molecule therapeutic agents targeting ARs exist, their use represents an emerging area, with most drug prototypes still in the pre-clinical developmental stage and many lacking large-scale clinical trials. The future lies in identifying improved AR synthetic ligands with enhanced efficacy and selectivity, as well as reduced adverse side effects, along with establishing a platform of specific and diversified pre-clinical tests, to inform in turn the resulting clinical investigations.

## 1. Introduction

**Figure 1:**
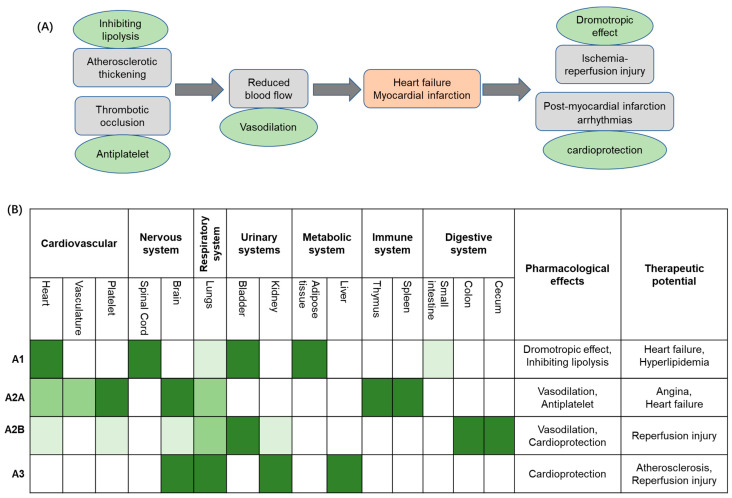
(**A**) The clinical context of ischemic heart disease (IHD) therapy. (**B**) Schematic overview of adenosine receptor distributions in various organs and tissues and therapeutic potential in the IHD context. Receptor physiological effects, pharmacological effects of ligands suitable for treatment, and their relevant clinical use are reported. White color: not found/known expression. Light to dark green colors: low, moderate, and high expression, respectively.

Ischemic heart disease (IHD) continues to rise disproportionately in low- and middle-income countries [1,2,3,4,5,6], whilst a decline is recorded in higher-income nations. Recent studies suggest that economic disparities between countries exacerbate health inequalities, amplifying the impact of IHD on vulnerable populations [1,2,6,7]. IHD incidence is projected to continue to rise, with the World Health Organisation forecasting it will rank among the top three causes of mortality, with an anticipated toll of 9.3 million in 2030 [7]. The United Nations 2030 Agenda for Sustainable Development Goals aims to reduce the premature mortality rate of ‘Non-Communicable Diseases’ by one-third, promoting the development of novel and effective therapeutic strategies to combat IHD and to mitigate its global impact [6]. Pathophysiologically, IHD (also referred to as coronary artery disease) is a condition characterized by reduced blood flow (i.e., ischemia) to the myocardium (with simultaneous imbalance between myocardial oxygen supply and demand), typically due to atherosclerotic narrowing (i.e., stenosis) or occlusion of coronary arteries due to the formation of plaques, which if undergoing rupture may cause thrombosis, resulting in myocardial infarction (MI) (Figure 1A). IHD’s current treatment paradigm encompasses a multifaceted approach aimed at alleviating symptoms and preventing acute events and disease development, thereby also enhancing overall cardiovascular function [8]. However, pharmacological management remains a cornerstone in the treatment protocols of IHD. Primary clinical strategies target platelet aggregation, plasma lipid concentration, plaque progression, thrombosis, regulation of blood pressure, and cardiac oxygen supply/demand in order to improve patients’ clinical outcomes, mitigate MI risk, and reduce morbidity and premature mortality [8,9]. Notably, antiplatelet agents, such as aspirin, are a foundational therapy in the context of IHD, crucially reducing thrombus formation in coronary arteries [10,11]. Similarly, purinergic P2Y12 receptor inhibitors, such as clopidogrel and prasugrel, have been extensively investigated in IHD treatment and their therapeutic properties widely adopted in clinical protocols [12,13,14,15,16]. Further, when combined with aspirin, these agents determine a more potent antiplatelet effect, which significantly decreases the risk of recurrent ischemic events in IHD patients [12,16]. With regard to the management of blood lipid concentration, statins such as atorvastatin and rosuvastatin have proven pivotal in IHD treatment through decreasing intracellular cholesterol levels [17] and, ultimately, leading to the stabilisation of atherosclerotic plaques, thus reducing cardiovascular event risk [18]. Lastly, anti-ischemic agents (e.g., β-blockers, Ca^2+^ channel blockers, and nitrates), angiotensin-converting enzyme inhibitors (such as enalapril and ramipril), and angiotensin II receptor antagonists (such as losartan and candesartan) have also been used in IHD patients [19,20,21], mainly to reduce blood pressure, as well as prevent cardiac remodelling and fibrosis [22]. In this context, the application of AR ligands represents an emerging research area to identify novel therapeutic opportunities, thereby enhancing cardioprotection effects in IHD.

Overall, this review aims to consolidate recent progress in the development of synthetic small-molecule AR ligands, emphasizing structurally diverse compounds with distinct receptor selectivity and tailored pharmacokinetics. This work uniquely highlights that some underexplored ligand classes may have therapeutic promise in cardiovascular contexts, with a primary focus on IHD. A significant contribution lies in the *in-depth* analysis of emerging small-molecule ligands based on (poly)heterocyclic scaffolds and their rational design to access an enhanced AR subtype selectivity, as well as to overcome efficacy and safety limitations of current drug prototypes. Ultimately, the review also offers a detailed inspection of a selection of widely investigated AR targeting molecules, supported by experimental and clinical outcomes, in order to endorse further a possible broadened scope for these agents (beyond traditional pharmacological use), including to serve as lead candidates in drug discovery programmes which focus on the identification of effective AR-targeted therapeutics in IHD.

## 2. Adenosine

Adenosine is an endogenous purine nucleoside synthesised from the dephosphorylation of ATP, ADP, and AMP via nucleosidases [23]. Adenosine exerts distinct roles within the body by targeting the so-called adenosine receptors (ARs), a sub-family of G-protein coupled receptors (GPCRs) classified into four subtypes, namely A_1_, A_2A_, A_2B_, and A_3_, thereby regulating various systems, such as cardiovascular, nervous, respiratory, and urinary systems (Figure 1B) [24,25]. Extracellular adenosine levels are meticulously regulated via enzymatic pathways and cellular transport systems, maintaining physiological equilibrium. Under basal conditions, adenosine is kept at low micromolar concentrations via equilibrative nucleoside transporters, which facilitate bidirectional flux across cellular membranes. In contrast, under pathological conditions such as ischemia, hypoxia, or inflammation, extracellular adenosine concentrations become dramatically elevated [26]. Elevation is primarily driven by the enzymatic breakdown of extracellular ATP and ADP to AMP, which is then dephosphorylated to adenosine by the ecto-5′-nucleotidase CD73 [27]. The resulting increase in adenosine determines a critical adaptive response, activating various protective signalling cascades that act by mitigating cellular injury and promoting tissue recovery.

Briefly, the high expression of A_1_ receptors (A_1_Rs) in the heart is closely linked to their negative dromotropic effect, making them a potential therapeutic target for heart failure. In adipose tissue, A_1_R is also highly expressed; its ability to inhibit lipolysis suggests therapeutic value in hyperlipidemia. The high expression of A_2A_ receptors (A_2A_Rs) on platelets underpins their anti-platelet aggregation effect, which reduces thrombosis and consequently lowers the risk of heart failure. Differently, the presence of A_2B_R in human platelets is currently under debate and is probably of minor function [28,29]. A_2A_R is also relatively abundant in the heart, where its vasodilatory effects offer therapeutic potential for angina pectoris. Although the expression of A_2B_ receptors (A_2B_R) in the heart is relatively low, they can still contribute to vasodilation and may thus play a role in the management of angina. The inhibition of A_2B_R and A_3_ receptors (A_3_R), despite their low expression levels in the cardiovascular system, has been shown to influence pro-inflammatory pathways that help mitigate cell death and tissue damage during ischemia-reperfusion injury.

## 3. Historical Perspective of AR Targeting and ‘Cardioprotection’

Early observations on the effects of AR ligands on the cardiocirculatory system date back to the twenty years between 1950 and 1970, when adenosine was first reported for its vasodilatory properties, especially in coronary circulation. In parallel, several studies also highlighted the rise of adenosine levels in ischemic myocardium, leading to vasodilation and protection [30,31]. Subsequently, in the 1970s, it was widely observed (and generally accepted) that the protective effects of this nucleoside extended beyond vasodilation, for instance affecting the heart rate, conduction, and inflammation [32]. Following the identification and characterization of the four AR subtypes (A_1_, A_2A_, A_2B_, A_3_) in the 1980s, led to an interest in assessing their potentially distinct cardiovascular roles [33]. Overall, A_1_R was investigated for anti-arrhythmic and negative chronotropic/dromotropic effects, A_2A_R for coronary vasodilation and anti-inflammatory properties, A_2B_R (which resulted in upregulation during ischemia) for possible continued cardioprotection in late stages, while A_3_R’s effects on cardiovasculature were less known in the first phases and mainly linked to potential preconditioning pathways [34,35,36]. During the 1990s, extensive preclinical studies were conducted to clarify the exact role of ARs in cardioprotection and ischemic preconditioning [31,37,38]. Adenosine was initially found to be involved in ischemic preconditioning, namely the process where ‘soft’ ischemia episodes protect against subsequent prolonged ischemic events. Furthermore, synthetic AR agonists were also widely investigated in this regard, demonstrating preconditioning-like effects in animal models, along with reduced infarct size and improved functional recovery. Relatively often, the systemic side effects, such as bradycardia (e.g., with A_1_R targeting) and hypotension (e.g., with A_2A_R targeting), were considered as evident main challenges associated with the use of AR modulators, along with narrow therapeutic windows in some cases, which make prolonged exposure difficult. Nonetheless, several clinical trials have been conducted (from the 2000s) to assess the suitability of administering adenosine (as well as synthetic ligands) during acute cardiovascular damage, such as during MI and ischemia–reperfusion injury [39], often leading to contrasting (or not univocal) outcomes. Some of these experimental programmes demonstrated improved myocardial perfusion and reduced infarct size [40], such as in the case of the AMISTAD I/II trials, suggesting that intracoronary or intravenous adenosine can be beneficial, particularly when administered in the early phases of the acute event [41,42,43]. Moreover, in the context of acute myocardial infarction (AMI), vasodilatory, anti-inflammatory, and anti-platelet effects, primarily through A_2A_R receptor activation, have also been considered relevant (as evidenced by the early results with the agonist Regadenoson, *vide infra*), although the study informed on the absence of significant mortality benefits overall. In a similar fashion, the REOPEN-AMI trial demonstrated improved microvascular perfusion post-coronary intervention [44], but no long-term outcome improvement, while the ATTACC study did not indicate an improvement with the use of adenosine in terms of cardioprotective clinical outcomes [43,45]. Other studies also failed to show significant benefit, often due to short plasma half-lives (e.g., seconds to minutes) and off-target effects.

Over the last 25 years, the development of synthetic, sometimes isoform-selective, small-molecule ligands has been the focus of extensive investigations aimed at improving the efficacy and reducing the hemodynamic side effects within therapeutic protocols based on the modulation of the AR system. Lastly, in more recent times, an increasing interest has been recorded in shifting the research focus towards studies on combination therapies, exploring AR targeting as an adjunct treatment to other therapies, for instance, to treat reperfusion injury or prevent cardiac remodelling post-acute myocardial infarction. To summarize, although AR ligands have shown consistent preclinical promise in reducing ischemic injury and modulating cardiac function, the translation to human therapies has been challenging. Decades of promising basic science on AR-mediated cardioprotection have, unfortunately, led to inconclusive clinical trial outcomes in general, highlighting that there is an ongoing translational gap between results in preclinical and human studies, which often represents one of the major challenges in cardiovascular medicine [46]. Undoubtedly, advances in receptor pharmacology (e.g., possibilities of biased and/or allosteric modulation of agonists), as well as the discovery of new agents and pharmaceutical formulations (e.g., isoform-selective ligands, prodrug approaches, myocardium-targeted delivery systems), continue to hold potential for novel treatments in IHD [42,47,48,49], especially for long-term cardiac care. In particular, newly developed AR-targeted compounds have the potential to overcome the therapeutic limitations of traditional AR ligands. Typically, these are low AR subtype selectivity and consequent off-target actions, poor pharmacokinetics (e.g., short half-lives), poor oral bioavailability, or instability in plasma, typical side effects (e.g., bradycardia for A_1_R agonists, increased heart rate and blood pressure for A_2A_R antagonists), as well as lack of functional selectivity (i.e., biased effects) enabling to differentially affect beneficial vs. adverse signalling pathways. With regard to this latter aspect, for instance, one such ligand could potentially promote cardioprotection while causing bradycardia as a side effect. Similarly, the identification of effective prodrugs and metabolically stable analogues could improve oral bioavailability and plasma half-life, thereby further contributing to preserving cardiac functions more effectively.

## 4. Basic Summary of Adenosine Receptors (ARs) and Cell Biology Implications

**Figure 2:**
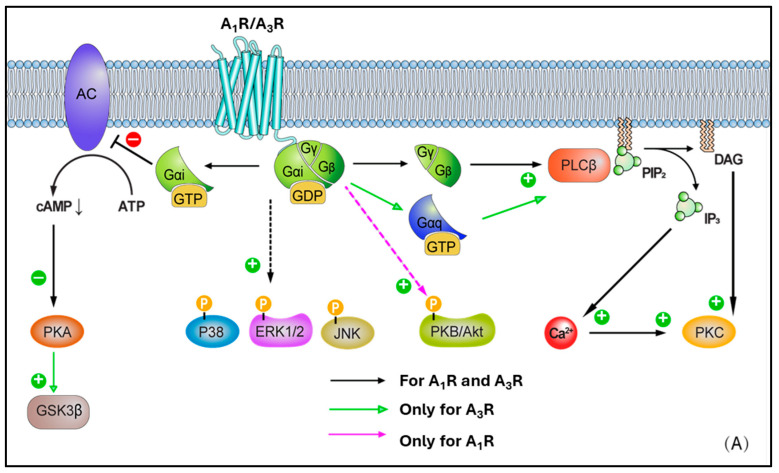

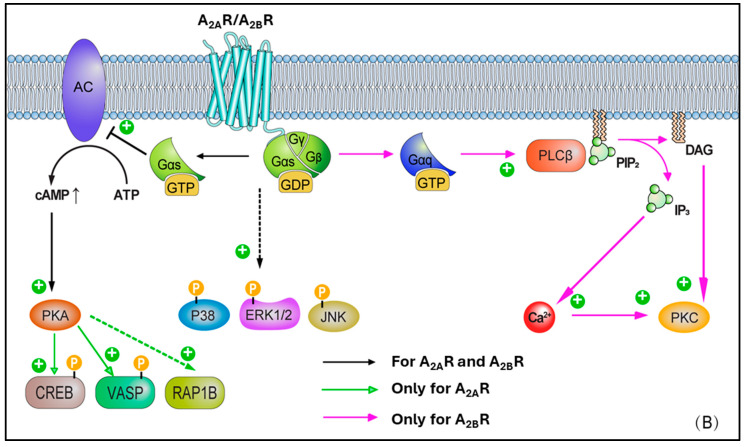
(**A**) Cell biology overview of A_1_R and A_3_R intracellular signalling pathways. (**B**) Cell biology overview of A_2A_R and A_2B_R intracellular signalling pathways.

The A_1_ receptor (A_1_R) is expressed throughout the human body. High levels are observed in the heart, adipose tissue, bladder, and spinal cord, while lower levels are found in the lungs, small intestine, smooth muscle cells, and coronary endothelial cells [24]. With regard to the heart, local A_1_R expression in the myocardium is not homogeneous, but with greater concentration in the right atria compared to the left. Adenosine possesses a high nanomolar affinity for the A_1_R, leading to activation. The A_1_R is linked via the G-protein Gi, determining reduced adenylate cyclase (AC) activity and lowering cAMP levels. Reduced cAMP levels prevent protein kinase A (PKA) activation. Further, released βγ subunits from Gi may affect voltage-gated calcium channels and activate phospholipase C (PLC) [50,51] (Figure 2A). Additionally, A_1_R activation has been found to affect the phosphorylation of p38 mitogen-activated protein (MAP) kinases, extracellular signal-regulated kinases 1/2 (ERK 1/2), and c-Jun N-terminal kinases 1/2 (JNK 1/2) [27]. These signaling pathways are closely associated with the regulation of inflammation, apoptosis, cellular stress responses, and cell proliferation. Modulating these pathways helps promote cardiomyocyte survival and repair, thereby reducing cell death and adverse cardiac remodeling following ischemic injury [52,53,54]. In the cardiovascular system, activation of A_1_R leads to a decrease in intracellular cAMP levels, which in turn enhances K^+^ efflux. This results in negative dromotropic effects by suppressing pacemaker activity at the sinoatrial node, His bundle, and atrioventricular node [24] (Table 1). Moreover, A_1_Rs have been shown to be in a clear relationship with lipolysis, whereby playing a substantial regulatory role in the pathway transforming triglycerides in adipose tissue into free fatty acids and glycerol [55,56,57,58]. Overall, A_1_R activation inhibits lipolysis. When adenosine binds to A_1_Rs on adipocytes, there is an inhibition of adenylyl cyclase, giving lower intracellular cAMP levels, which in turn produces a reduction of the activity of PKA. This latter is crucial for activating hormone-sensitive lipase (HSL), a main player enzyme responsible for the lipolysis process, which is reduced with A_1_R activation (agonism). In contrast, A_1_R antagonists (and/or blockers) have been found to promote lipolysis, raising the interest in their use to treat obesity (via weight-loss therapies), or some metabolic diseases, and to manage conditions like type 2 diabetes [55,59,60]. A smaller number of studies have also reported that A_2A_R and A_2B_R subtypes could have a role in the process under some conditions [61,62,63,64], although this remains largely less characterized at the present time. Lastly, there is also initial evidence that A_1_R antagonism could produce beneficial effects on LDL (low-density lipoprotein) and HDL (high-density lipoprotein) levels. In this regard, animal studies have examined the use of adenosine A1 receptor antagonists, highlighting that the use of these agents could lead to a favourable ratio LDL/HDL, with an increased concentration of the latter and, in parallel, a reduced total cholesterol and triglycerides in some cases. [62,65,66,67]

A_2A_ receptor (A_2A_R) expression is greatest in the thymus, brain, spleen, platelets, and intermediate in the lung, vasculature, and heart [68]. Within this latter, A_2A_Rs are particularly expressed in the atria and ventricular tissues. Unlike the A_1_R, A_2A_R is linked to the G protein Gs and, upon activation, this leads to stimulation of adenylate cyclase (AC) activity, higher cAMP levels, and PKA activation. In turn, this latter stimulates Ras-related protein Rap-1b (RAP1B) and vasodilator-stimulated phosphoprotein (VASP), both highly relevant in the context of vascular cells and platelets (as targets of PKA), as well as cAMP-responsive element binding protein (CREB) phosphorylation (Figure 2B). CREB activation promotes the expression of anti-apoptotic genes such as Bcl-2, upregulates anti-inflammatory molecules like IL-10 and HO-1 to mitigate inflammation-induced secondary injury after ischemia-reperfusion, and enhances the expression of angiogenic factors such as VEGF to support vascular regeneration and functional recovery in ischemic myocardium [69,70,71]. Protein kinase B (PKB or Akt) and p38 MAP kinases, ERK 1/2, and JNK 1/2 phosphorylation induced by A_1_R can be further enhanced following A_2A_R activation, as has been shown in neural tissues [72]. This represents a classical example of receptor crosstalk, where engagement of one receptor subtype influences signalling cascades typically associated with another, suggesting that a synergy in AR activation may be present, contributing to the observed phosphorylation events [73]. More in detail, this cross-talk was characterised in CHO and native vascular smooth muscle cell models, highlighting that A_2A_R activation elevates cAMP, leading primarily to PKA activation. In turn, the elevated cAMP/PKA activity can facilitate the phosphorylation of A_1_Rs or their downstream effectors, therefore effectively triggering the activation of A_1_-mediated pathways [74,75]. Through such modulation, ‘A_1_R-primed’ phosphorylation of PKB/Akt, p38, ERK 1/2, and JNK 1/2 can become more pronounced or, potentially, even happen only after prior A_2A_R activation. In such circumstances, A_2A_R may exert a temporal and/or hierarchical control on the activation of A_1_R–mediated signalling. Moreover, in ventricular myocytes, the activation of A_2A_R subtype has been reported to lead to inotropic effects [68], with the inhibition of voltage-gated calcium channels and L-type Ca^2+^ currents. Ultimately, this results in vasodilation via activation of endothelial nitric oxide synthase (eNOS), which converts L-arginine to nitric oxide (NO), and by opening ATP-dependent K^+^ channels, which determines K^+^ to exit the cell with following membrane hyperpolarization [24,27,68,76,77,78,79]

Differently to the other AR subtypes, A_2B_ receptors (A_2B_Rs) possess the least affinity for adenosine (in the micromolar range, e.g., 15–24 μM) [27]. However, this property may be selectively modulated by protein kinase C (PKC) signals [80]. High expression of A_2B_R is observed in the colon, bladder, and cecum, moderate expression is apparent in the eye and lungs, while low expression is found in the brain, kidney, and heart [81]. In smooth muscle, A_2B_R has been found to mediate vasodilation via Gs-stimulated adenylate cyclase (AC) activity, cAMP production, and PKA phosphorylation [82] (Figure 2B). A_2B_R can also activate phospholipase C-β (PLC-β) and increase Ca^2+^ ions intracellularly, in smooth muscle cells as well as in other cell types (e.g., vascular endothelial cells). Moreover, phosphorylated mitogen-activated protein kinases, p38, ERK 1/2, and JNK 1/2, resulting from A_1_R activation, are further activated following A_2B_R recruitment. In a similar fashion to A_2A_R, A_2B_R activation leads to vasodilation via nitric oxide and potassium ATP channels [81,83,84,85,86], in various cell types, including endothelial cells, ventricular myocytes, and vascular smooth muscle cells, although this appears to be produced to a lesser extent.

Lastly, the A_3_ receptor (A_3_R) is the most recently identified AR subtype, which exhibits the most variable expression pattern across species. Although non-human A_3_Rs show little affinity to methylxanthine antagonists (e.g., caffeine and theophylline), in humans, the A_3_R is xanthine sensitive. The relatively high affinity of A_3_R for methylxanthines has pharmacological and physiological relevance, particularly in the context of drug development and therapeutic interactions, as the receptors are blocked by the common assumption of these xenobiotics commonly present in dietary intakes [87]. A_3_R is widely expressed in the human body, with lungs and liver being the main sites, although relatively high presence has been recorded in other tissues such as the brain and kidney [24]. Following activation, the A_3_R-mediated intracellular signalling results in Gi-mediated decrease in adenylate cyclase (AC) activity and reduced cAMP production, activation of glycogen synthase kinase-3β (GSK-3β) and, consequently, the decrease in β-catenin, cyclin D1, and c-Myc transcription factor (Figure 2A). Active (dephosphorylated) GSK-3β promotes the opening of the mitochondrial permeability transition pore (mPTP), leading to mitochondrial membrane potential loss and cytochrome C release, which triggers cardiomyocyte apoptosis and necrosis. GSK-3β activation is a key driver of myocardial injury during ischemia-reperfusion, and its inhibition has been demonstrated to confer cardioprotective effects [88,89,90]. Additionally, A_3_R can couple to G_q_/_11_ and determine the activation-mediated increase in PLC-β and Ca^2+^, as well as RhoA and phospholipase D (PLD). Overall, A_3_R expression in the myocardium is reported to be low [24,81]. Within the heart, this subtype has been observed to play a role in smooth muscle cells (in addition to the effects on coronary artery muscle cells) [24,91,92], where it can lead to vasoconstriction, especially under ischemic or inflammatory conditions, despite the limited evidence of expression in such sites.

## 5. AR Cross-Talks with Other Signalling Pathways

Adenosine receptor signalling intricately intertwines with multiple other cellular pathways, producing a fine-tuned regulation of cellular processes. Indeed, these cross-talks allow adenosine to exert its effects in context-dependent manners, influencing a diverse range of physiological functions [93]. For instance, this is seen in the interaction between adenosine A_2A_Rs and receptor tyrosine kinases (RTKs) in several cell types, including cardiomyocytes, cardiac fibroblasts, and vascular endothelial cells [94,95]. Collectively, these two receptor systems modulate cell growth, differentiation, and survival, thereby influencing processes such as angiogenesis and tissue repair. Furthermore, A_3_R activation is known to be particularly noteworthy in cancer, for modulating apoptotic and anti-proliferative pathways [96]. A_3_R signalling promotes apoptosis in tumour cells, counteracting pro-survival signals from RTKs and growth factor receptors, ultimately inhibiting tumour cell proliferation. In this regard, A_3_R is often overexpressed and upregulated in tumour cells [97], but not in normal tissues, its inhibitory effect on tumour growth is likely due to a combination of factors, such as inhibition of PI3K/Akt, activation of JNK and p38 MAPK, inhibition of NF-κB, mitochondrial-mediated apoptosis, ROS generation, and cell cycle arrest [97,98,99,100,101,102]. In a similar fashion, AR signalling has been demonstrated to interact with Toll-like receptors (TLRs) in order to regulate the activation of immune cells [103], thus contributing to fine-tuning the immune response, particularly in preventing excessive inflammation. This crosstalk is found to be mainly characterized by the suppression of TLR-induced pro-inflammatory signalling (further influenced by increased IL-10 production) via A_2A_R, as well as a modulatory action via A_2B_R, for instance during hypoxia events in inflammation [104,105,106], although A_1_R and A_3_R have also shown some level of interaction with TLRs [107,108]. This crosstalk is particularly important in immune responses to both inflammation and infection, where adenosine is known to truncate excessive activation of immune cells via A_2A_Rs, while also enhancing tolerance-promoting effects of TLRs. In parallel, adenosine is also widely considered an established and pivotal regulator of immune responses, with its effects modulated via receptor subtypes, respective cross-talks, as well as the nucleoside’s extracellular concentration [23]. All these are reported to be fundamental to maintain immune homeostasis and prevent tissue damage from uncontrolled inflammation [93]. In this regard, it is known that adenosine suppresses excessive inflammatory damage under physiological conditions, ensuring responses are proportional to stimuli. This is largely achieved through participation by A_2A_Rs, which are expressed in several immune cells (Table 2), including dendritic cells and T lymphocytes [93]. Gs-coupled A_2A_R stimulation increases intracellular cAMP, thereby triggering anti-inflammatory signalling cascades, including the upregulation of the cytokine IL-10, secreted by macrophages, T cells, and dendritic cells. In turn, IL-10 contributes to the activation of macrophages, which is essential for the resolution of inflammation, as well as to the polarization of these immune cells toward an anti-inflammatory phenotype, which is associated with plaque repair and stability during atherosclerosis processes [109,110]. Based on this, the A_2A_R/IL-10 axis is considered a potential main player to preserve cardiac functions, also in the case of MI remodeling, involved in the reduction of infarct size, improvement of ventricular functions, and decrease in long-term incidence of heart failure [111,112,113].

However, adenosine’s immunosuppressive effects have been demonstrated to possess dual implications. Specifically, if in the tumour microenvironment elevated adenosine levels, fuelled by hypoxia and upregulation of ectonucleotides (such as CD39 and CD73), play an important role in immune evasion (primarily, through A_2A_R-mediated actions) [114]. At the same time, elevated adenosine concentrations are reported to inhibit the activity of cytotoxic T lymphocytes (CTLs) and natural killer (NK) cells, which are vital for anti-tumour immunity [114]. In contrast, it has also been found that A_2A_R on dendritic cells and macrophages shifts their phenotype toward immunosuppression. In turn, this effect could create an optimal environment for metastasis and tumour growth [114]. Furthermore, in chronic inflammatory diseases such as rheumatoid arthritis, adenosine’s immunosuppressive properties can be exploited to be therapeutically advantageous [115]. For instance, methotrexate (a cornerstone drug for rheumatoid arthritis) is believed to exert part of its efficacy through extracellular adenosine level increases. Subsequently, adenosine accumulation is found to reduce joint inflammation and promote tissue repair principally via A_2A_R-mediated crosstalk with anti-inflammatory pathways (although A_3_R can also be involved in this context) [115]. Similarly, in sepsis-controlled modulation of adenosine, direct signalling and cross-talks can protect tissues by curbing excessive inflammatory responses and mitigating oxidative damage [116]. Overall, this intricate signalling network indicates that adenosine has a key impact in finely influencing cellular responses, in both normal and pathological contexts.

## 6. AR Synthetic Small-Molecule Ligands

Adenosine’s therapeutic effects have been well-documented in the literature for a long time [117,118], as well as the evidence of actions (sometimes contrasting) towards cardiocirculatory diseases, including IHD [30,34,80,119,120,121,122,123,124,125,126], especially in acute settings due to the known vasodilatory, anti-inflammatory, and antiplatelet properties. However, adenosine’s short half-life renders the clinical actions short-lived; therefore, the use of the endogenous nucleoside as a drug is currently considered unachievable or, at least, highly debatable, especially for chronic conditions [24,127]. Adverse drug reactions can also be present with adenosine due to the widespread receptor expression, along with the non-selective isoform binding profile, which is additionally considered to trigger a variety of side effects [24,117]. This has prompted the exploration of AR synthetic ligands, mainly heterocyclic small molecules, which can possess improved receptor selectivity, potency, and bioavailability, thus enabling more efficient and targeted therapeutic effects, along with a minimized chance of generating side reactions.

AR ligands are mainly classified as agonists, antagonists, or partial agonists [23,24,25], although terms producing biased effects have also been recently reported [128]. Each of these may offer distinct therapeutic benefits (see Figure 1B). Namely, agonists mimic adenosine’s action by activating receptors, for instance, giving benefits in reducing inflammation, as well as enhancing myocardial perfusion. Antagonists, on the other hand, block receptor activity, which may be advantageous in specific settings to counteract excessive endogenous adenosine signalling. Partial agonists and biased agonists may offer a balanced approach, providing moderate or partial activation of receptors. Biased effects refer indeed to the preferential influence on only one/a limited number of signalling pathways linked to such a receptor, which in turn can potentially minimise adverse effects whilst preserving therapeutic efficacy. In the context of this review, AR-targeted biased ligands can allow for fine-tuning therapeutic approaches for IHD, ischemia-reperfusion injury, heart failure, and myocardial infarction [129,130,131,132,133]. For instance, cardioprotective actions with A_1_R-biased agonists could promote anti-ischemic effects (e.g., reduced Ca^2+^ overload, improved mitochondrial function) without inducing bradycardia or atrioventricular block. Differently, A_2A_R biased agonists could enhance anti-inflammatory or vasodilatory pathways without triggering hypotension or immunosuppression. Lastly, A_3_R biased ligands may reduce infarct size by promoting mitochondrial protection and preventing apoptosis, while avoiding systemic side effects.

**Figure 3:**
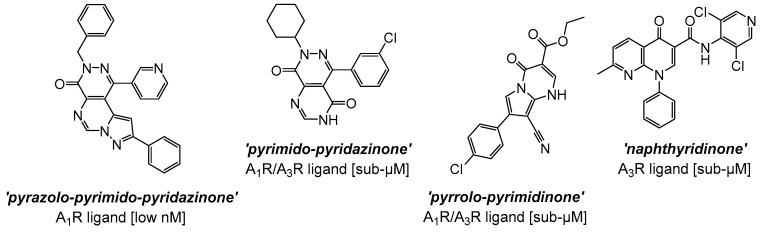
Selected hit compounds based on poly-heterocyclic structures developed as AR ligands, resulting in high potency and/or selectivity profiles as antagonists.

Recent advances in the development of synthetic small-molecule AR ligands have focused on accessing newer and diverse heterocyclic compounds, mostly based on non-purine polycyclic cores [134]. These fulfil classical structural requirements known to determine robust and extended interactions with ARs, such as the highly planar nature, given by the presence of fused sp^2^ hybridized aromatic rings, or π-electron rich and poly-*aza*heterocycles [135]. The adoption of a variety of ‘adenosine-derived’ or ‘adenine-like’ bicyclic and tricyclic systems in the design of new analogues, to mimic binding affinities and/or properties of adenosine, has led to the development of AR binders with exceptionally high affinity and selectivity [134,135,136]. For instance, early hits based on tricyclic structures developed as AR ligands belong to the pyrazolo-pyrimido-pyridazinone series [136,137,138], one example of which is shown in Figure 3, demonstrating pronounced antagonist activity (e.g., in the low nanomolar range), as well as relevant selectivity toward the human A_1_R subtype (e.g., approximately 100-fold over the A_2B_R). Structural activity relationship (SAR) studies conducted on this class of tricyclic analogues indicate that: (i) the pyrazole ring is essential to shift the selectivity towards the A_1_R; (ii) the nature of the substituents (e.g., aromatic *vs*. aliphatic) at the pyrazole 1-position plays also a crucial role for A_1_R affinity, particularly in the case of heteroaromatic substitutions introduced in this position; (iii) the introduction of bulkier hydrophobic groups (e.g., benzyl or *t*-butyl) in the pyrazole ring can increase A_1_R potency, possibly via additional lipophilic interactions in the receptor pocket; (iv) electron-donating groups on aryl ring/s may also increase potency, possibly via enhanced π-stacking or hydrogen bonding with amino acidic residues in the binding site; (v) modifications of the substituents (e.g., methyl, amino) in the pyrimidine moiety can enable to fine-tune selectivity [136,139]. Overall, the development of these poly-*aza*heterocyclic series confirmed that planarity (i.e., mimicking purine), hydrogen bond acceptor capability, and suitable orientation of substituents into the binding pocket are all essential features to take into account to access potent, high-affinity, and isoform-specific small-molecule ligands targeted to ARs.

Subsequently, refinements of the tricyclic structures have been carried out on this series [140], via molecular simplifications strategies mainly focusing on (1) the removal of one aromatic/heterocyclic ring in the scaffold (a particular example being the pyrimido-pyridazinone A_1_R/A_3_R mixed analogue, where the elimination of the pyrazole ring i.e., a critical element for A_1_R activity in the tricyclic series, was performed; Figure 3) and, simultaneously, (2) the alteration of the pattern of nitrogen inclusion in the heterocyclic system (see, as examples, the pyrrolo-pyrimidinone A_1_R/A_3_R mixed antagonist and the naphthyridinone analogue, which is a preferentially selective A_3_R antagonist; Figure 3). [140]. Overall, SAR considerations on these series [98,99,100,101,136,137,138,140] suggest that both tricyclic and bicyclic fused *core* systems are capable of mimicking adenosine’s flat purine-like structure, thereby likely engaging in key hydrogen bonding and π-π interactions within the orthosteric binding site. Nonetheless, the removal of the pyrazole ring, or the simplification of the polyheterocyclic structure (i.e., from tricylic to bicyclic) result in generally lower activities, likely due to the reduced rigidity and/or extended planarity, loss of key hydrogen bonding and *π-π* interactions, which in turn may lead to a suboptimal fit in the binding pocket of the receptor.

**Figure 4:**
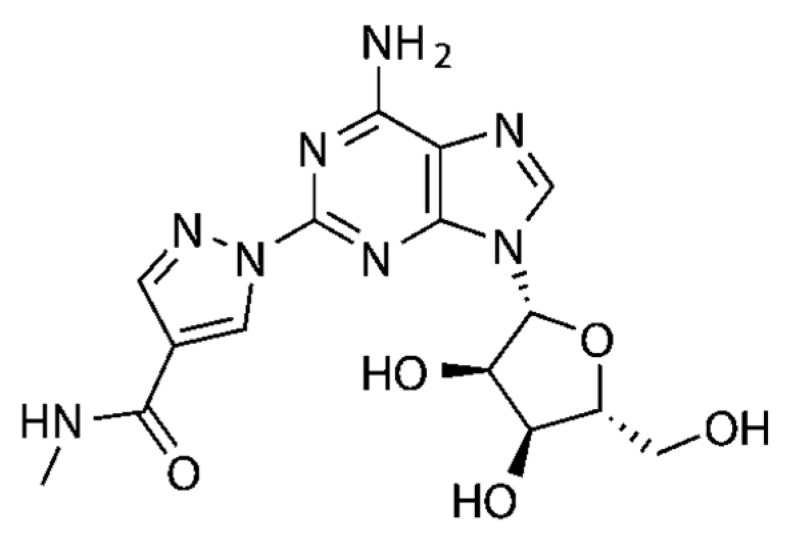
Chemical structure of the A_2A_R small-molecule agonist regadenoson (marketed under the brand names of Rapiscan^TM^, by GE HealthCare, and Lexiscan^®^, by Astellas Pharma US, Inc., Northbrook, IL, USA) used as an imaging adjuvant in radionuclide myocardial perfusion imaging.

In a similar fashion, several series of polycyclic ligands were developed through similar molecular drug design and synthetic approaches [134,135,139,140,141,142,143,144], where substitutions at defined positions on the heterocyclic scaffolds were systematically explored to optimise binding affinity and selectivity towards AR isoforms, which were mostly recorded via radioligand binding assays and/or fluorescence-based readouts [134,135,136,137,138,139,140,141,142,143,144,145]. These early findings are encouraging, suggesting that small-molecule synthetic ligands may offer valuable opportunities as alternative drug candidates for AR therapeutic targeting. In line with this, it would appear that the development of AR small-molecule ligands with improved pharmacokinetic properties has the potential to address key gaps in IHD management, ultimately advancing the therapeutic landscape for cardiovascular diseases [146,147]. In the context of cardioprotective benefits, these can lead to the identification of new agents which could expand the array of drugs in use, or can be employed as adjunct therapeutics to current treatment protocols and diagnostic procedures [76,139,148]. For instance, the latter is the case of regadenoson (Figure 4), an A_2A_R agonist that causes coronary vasodilation (followed by quick hyperaemia, maintained for a relatively extended time) and which has been approved for clinical use in pharmacologic stress testing during myocardial perfusion [149]. Regadenoson use is suitable for effective radio-imaging procedures, such as in the case of single-photon emission computed tomography (SPECT), cardiovascular magnetic resonance (CMR), computerized tomography CT), and positron emission tomography (PET) scans.

## 7. Current Gaps in Clinical Translation of AR Ligands

Despite significant progress in developing AR small-molecule ligands, gaps remain in research and development regarding their long-term safety and efficacy, ahead of assessing beneficial application in IHD. Existing studies mainly focus on the acute use of AR-targeted compounds during ischemic events [80,125,126,128,146], as cardioprotection from adenosine has been considered to be highly timing-dependent–i.e., most of the action occurs when adenosine is administered just before or during the early phases of an AMI event (e.g., <5–10 min window) [150,151,152,153]. However, in some clinical trials, the actual experimental logistics led to delayed administration, possibly reducing the efficacy [154,155,156]. Additionally, less is known about the safety and effectiveness of AR ligands for chronic use, as it would be important for continued management therapies. Many active compounds still exhibit puzzling therapeutic outcomes, increased adverse event rates in patients with compromised cardiovascular function, and other variable systemic and/or off-target effects (e.g., bradycardia, atrioventricular block with A_1_R activation; hypotension, tachycardia, immunosuppression with A_2A_R/A_2B_R activation) [154,157,158]. The latter are mainly attributable to the lack of isoform selectivity of AR ligands clinically tested for cardioprotective effects, which often led to determining positive cytoprotective effects mixed with detrimental hemodynamic changes. Studies investigating prolonged administration are limited, and unanswered questions remain regarding tolerance risk and adverse effects profile with long-term receptor-positive stimulation or inhibition. Furthermore, while animal studies have shown promising cardioprotective effects for a number of AR ligands [80,134,146,157], the transition to human trials is yet to be fully explored. Differences in adenosine receptor distribution and function across species may contribute to these discrepancies, highlighting the need for additional therapeutic research to confirm the pre-clinical findings, thus enabling translation to human use. Moreover, in vivo preclinical studies typically use young, healthy small animals (e.g., rodents) modelled with controlled ischemia-reperfusion protocols. This may represent a completely different context in comparison to human patients, being presented with comorbidities (e.g., diabetes, hypertension, atherosclerosis), parallel therapeutic plans, and age-related changes, which can also alter receptor expression or signalling. Overall, the development of highly selective AR synthetic small-molecule ligands remains challenging. In this regard, rational drug design and structural improvements of current prototypes may play a critical role in this research field, leading to the identification of novel pharmacophores and/or more effective agents, as required to enhance receptor specificity and minimise side effects [158,159]. Addressing these gaps will be crucial to leverage the full therapeutic potential of adenosine-targeted treatments in IHD, as well as in cardiocirculatory disorders in general.

## 8. Discussion

Adenosine’s lack of selectivity for receptor subtype and the poor half-life (when exogenously administered) has catalysed the development of synthetic analogues [134,144,146] with expectations of greater bioavailability and biodistribution profiles. Initial research on the development of AR synthetic ligands had been carried out as early as the 1980s. Hosey et al. were among the first to report on the inhibitory effects of *N*-6-phenylisopropyladenosine [160], namely a synthetic adenosine analogue, which has since become a widely used pharmacological tool to investigate AR functions. After Hosey’s seminal study, AR synthetic ligand research has expanded greatly, with a multitude of studies exploring both therapeutic and diagnostic applications in a variety of medical conditions [27]. Over the last 40 years, research into the synthesis and biological evaluation of new AR synthetic ligands has surged exponentially [144], fuelled by the dynamic nature of adenosine receptors and their crucial role in physiological regulation, particularly within the central nervous, cardiovascular, and immune systems. This has positioned ARs as focal points for therapeutic development in an array of conditions, including neurodegenerative disorders [161], neuropathic pain [162,163], cancer [100], and IHD [80,126,146,164]. As a result, numerous trials have evaluated the therapeutic potential of AR synthetic ligands [165,166], particularly to treat neurological, cardiovascular, and inflammatory diseases [146,164,165,166,167] and, more recently, cancers [168]. These clinical programmes have evaluated AR synthetic ligands over diverse dosing regimens (as informed by earlier preclinical studies), sample populations, and outcomes, contributing to an evolving understanding of the therapeutic benefits and limitations of adenosine-based treatment in these diseases. As an example, Neladenoson bialanate (BAY 1067197) is an A_1_R partial agonist which demonstrated positive effects in the treatment of heart failure in animal models, was investigated in a phase 2 clinical trial as the pharmacological agent for the same condition [47]; however, the beneficial cardiac effects have thus far not been confirmed in patients [169]. Capadenoson (BAY 68-4986, also an A_1_R partial agonist) is a further example of an AR ligand tested therapeutically in the context of heart failure and other cardiocirculatory diseases such as angina and atrial fibrillation [47,170]. This agent, designed to provide beneficial actions of adenosine agonism without causing the detrimental effects associated with full A_1_R agonists (e.g., bradycardia and atrioventricular block within the heart), demonstrated promise in animal models, which again were not fully reproducible in trials with patients [171]. Primarily, capadenoson failed to show sufficient efficacy in human clinical trials (e.g., failed to significantly show an improvement of the left ventricular function, as well as a marked reduction in mortality or hospitalization). Although not approved as a drug, this agent remains a widely used reference pharmacological tool in AR research.

Nonetheless, a significant proportion of AR synthetic ligand experimental programmes remain in the preclinical stage, highlighting that further research is crucially needed, especially on the involvement of AR in the cardiovascular context, as well as realistic effects produced by AR ligands to inform potential therapeutic applications for IHD. This, in turn, will be useful to conclusively establish the effective efficacy of AR synthetic ligands to tackle this disease. Given the relevance of AR ligands as possible therapeutic tools for cardiovascular conditions, we explored a selection of promising synthetic small-molecule agents (developed from 2000 to date), namely, RPR749 [172], LJ-1888 [173,174], IB-MECA [175], GS-9667 [176], LassBio-294 [177], cyclopentyladenosine (CPA) [178], and PSB-15826 [179] (see structures in Table 3), in order to highlight novel treatment opportunities in the context of IHD. These agents are AR-targeted molecules, some of which were previously trialled for safety, efficacy, pharmacodynamic, and pharmacokinetic profiles clinically. More in detail, based on the increasing attention given to the identification of novel and more effective drugs for IHD, this review attempts to provide detailed information on challenges that can influence the clinical transition and outline areas for further investigation. In this regard, therapeutic benefits and current limitations of the selected drug candidates are evaluated in order to help establish how their possible inclusion in clinical protocols may enhance the IHD treatment landscape.

### 8.1. RPR749 (A_1_R Agonist)

Shah and co-workers [172] investigated the pharmacokinetics, pharmacodynamics, and safety profile of RPR749, a highly selective A_1_R agonist (with a potency reported to be in the low nanomolar range, while only negligible activity is found at A_2_A, A_2_B, or A_3_ subtypes [181]). This agent exhibited lipid- and triglyceride-lowering activity in healthy subjects. This is mainly due to the capability of inhibiting lipolysis in adipocytes. When activated by the agent, A_1_Rs inhibit adenylyl (via G_i_ coupling), leading to lower cAMP levels, decreased PKA activity, and reduced phosphorylation of various lipolytic enzymes. Consequently, this produces the suppression of lipolysis, leading to decreased plasma free fatty acid levels, which in turn determine a lower substrate availability for hepatic triglyceride synthesis, along with a reduction of very low-density lipoprotein and triglyceride levels in the circulation in the long term [172,182].

The clinical trial included 48 healthy male participants divided into six parallel dosing groups (5, 15, 60, 120, and 200 mg). A key strength of this study was the rigorous double-blind, placebo-controlled, parallel group, randomised design, which minimised bias and ensured reliable comparisons across treatment groups. Pharmacological results demonstrated that RPR749 was safe and well-tolerated, with no serious adverse events reported (at single oral doses up to 200 mg) [172]. The drug exhibited effective absorption, with maximum plasma concentrations approximately 30 times higher than those of its metabolite, RPR772, indicating a favourable pharmacokinetic profile. Additionally, the reported mean terminal half-life of 16.4 h suggests the potential for sustained pharmacological effects. Most notably, blood free fatty acid levels decreased significantly across all treatment groups, especially at higher doses, indicating RPR749′s pharmacological efficacy in reducing circulating levels of free fatty acids [183]. However, while mean serum levels of insulin, triglycerides, glycerol, and glucose remained within normal ranges, fluctuations were observed, particularly at higher doses (i.e., 200 mg dose group). These variations raise concerns about potential metabolic effects, which were not further explored in detail, as well as whether any effect was present with regard to low- and high-density lipoproteins (LDL, HDL), or total cholesterol. Therefore, while there is evidence of safety (i.e., no significant or clinically relevant cardiovascular adverse events, such as heart rate reduction, was observed at any dose tested) and favourable pharmacokinetic properties of RPR749 [157,172,183], it appears necessary to conduct larger clinical research studies to confirm long-term safety and potential metabolic impacts associated with the use of this A_1_R agonist. Undoubtedly, the effect of free fatty acid reduction represents a benefit that may facilitate the progress of RPR749 as a therapeutic agent in IHD.

### 8.2. LJ-1888 (A_3_R Antagonist)

The selective A_3_R antagonist LJ-1888 (i.e., (2R,3R,4S)-2-[2-chloro-6-(3-iodobenzylamino)-9H-purine-9-yl]-tetrahydrothiophene-3,4-diol; Ki ≈ 10–30 nM on A_3_R, while much less potent on all the other AR subtypes, i.e., >1 μM [142,173]) was first developed by da Jeong et al. [180] and, subsequently, explored in an in vivo study for its effects on atherosclerosis and hypercholesterolemia [173,174], using apolipoprotein E knock-out mice as an established model of atherosclerosis. The treatment with LJ-1888 was demonstrated to reduce atherosclerotic plaque formation and total cholesterol levels significantly, in comparison to control mice. These results suggest that LJ-1888 shows promise as a potential treatment for ischemic heart disease (IHD). However, further investigation is needed to fully understand its underlying pharmacological mechanisms. Long-term studies are also necessary to assess its efficacy and safety over time. In addition, it is important to evaluate any off-target effects or [185–188 multiple pharmacological actions, as these could complicate the interpretation of results in humans and potentially cause adverse effects [142,173,184]. Ultimately, further research will help determine whether the promising preclinical findings can be successfully translated into clinical trials. If so, LJ-1888 could contribute to improving the management of atherosclerosis and hypercholesterolemia in the context of IHD.

### 8.3. IB-MECA (A_3_R Agonist)

Auchampach et al.’s study investigated the cardioprotective effects of IB-MECA (also known as CF101 or Piclidenoson) [175], an A_3_R agonist with weaker activity on A_1_R and A_2A_R (IC_50_ ≈ 0.78–1.2 nM and Ki ≈ 1.1 nM on A_3_R, while Ki ≈ 54/56 nM on A_1_R/A_2A_R, respectively [185,186,187,188]). The study used a canine model of myocardial ischemia and reperfusion injury. Here, regional myocardial blood flow was evaluated, and myocardial infarct size was assessed through macro histochemical staining. The study provided a comprehensive evaluation of IB-MECA efficacy in mitigating tissue damage and preserving perfusion in acute conditions. The results revealed that IB-MECA with a single dose pre-treatment reduced infarct size by 40% compared to control groups, with similar reductions also observed when IB-MECA was administered during reperfusion [175]. The study noted no significant changes in hemodynamic parameters (such as heart rate, aortic pressure, and coronary blood flows) across treatment groups, suggesting the cardio-protection observed was independent of systemic hemodynamic alterations or alterations in blood flow. In subsequent investigations, IB-MECA and analogues (e.g., Cl-IB-MECA [189,190,191,192]) were additionally tested in mouse and rat in vivo studies [32,193], demonstrating a marked reduction of the infarct size when administered (i.v.) five minutes before reperfusion, or at the onset of reperfusion, respectively. This effect was abolished by A_3_ receptor antagonists, further supporting that the action is delivered via A_3_R-mediated signalling pathways. As all the studies above were at the pre-clinical stage, further research should be conducted to evaluate IB-MECA efficacy in humans, along with the assessment of longer-term treatment effects. However, the studies provide a preliminary foundation regarding the therapeutic potential of IB-MECA and inform on the suitability of investigating clinical translational opportunities. In addition, promising in vitro and in vivo results regarding IB-MECA have also been reported in cancer therapy [194,195,196], further propelling its promise as a therapeutic candidate, but larger, more extensive clinical trials are needed to extend these findings, as well as in the field of IHD treatment.

### 8.4. GS-9667 (A_1_R Agonist)

Initial in vivo investigations on GS-9667 (originally known as CVT-3619), a partial agonist for A_1_R (IC_50_ ≈ 6 nM on AC inhibition in adipocytes, via A_1_R [197]; Ki ≈ 55 nM on A_1_R, with selectivity over the A_2A_R > 200-fold, A_2B_R > 1000-fold and A_3_R > 20-fold [198]), highlighted its clinical potential as a therapeutic agent for IHD by effectively reducing plasma free fatty acid levels [196,199], without significant side effects in rodents. This result was also confirmed in human studies, where GS-9667 demonstrated significant efficacy in reducing free fatty acid levels in both non-obese and obese subjects, without inducing desensitisation or rebound effects during 14 days of treatment [176]. Notably, this effect was dose-dependent, with efficacy seen at oral doses as low as 300 mg. The rapid absorption and linear pharmacokinetics observed for the drug add further promise for its therapeutic use. The study’s design incorporated both single and multiple ascending dose trials in healthy volunteers, which established a strong safety and tolerability profile. Moreover, GS-9667 was well tolerated across all doses tested, with no significant side effects reported [176]. Despite the multiple strengths, the limitations of this study warrant attention. Predominantly, whilst the sample size (i.e., 88 participants) was sufficient for preliminary findings, it limits the generalisability of the results obtained, particularly in relation to long-term outcomes and effectiveness in a broader population. This aspect, along with the possible risk of interfering with bone homeostasis (where ARs expressed on osteoblasts, osteoclasts, and immune cells are known to influence various processes, including bone formation, differentiation, resorption, repair, as well as protective mechanisms) [183,200,201,202], stress the need for expanded trials to assess long-term safety, efficacy, and practical application in diverse patient groups, which could, however, demonstrate a positive role for GS-9667 in the treatment of IHD.

### 8.5. LassBio-294 (A_2A_R Agonist)

The therapeutic potential of LassBio-294 as a moderately potent A_2A_R agonist (i.e., µM EC_50_ values) has been recorded in a sodium-dependence shift assay and other functional tests in cardiac tissue [203,204]. This agent originally belonged to a class of drugs developed as phosphodiesterase inhibitors [205,206]. LassBio-294 was evaluated by da Silva et al. [177] with the aim of mitigating cardiac dysfunction associated with MI in spontaneously hypertensive rats. The findings revealed that LassBio-294 significantly improved cardiac function and prevented pathological remodelling in the MI rat groups, in line with earlier in vivo studies [207,208]. Moreover, ECG data showcased overall restored early mitral inflow velocities and enhanced exercise tolerance, indicating improved functional capacity [177]. LASSBio-294 also prevented the increase in left ventricular end diastolic pressure and enhanced sarcoplasmic reticulum calcium ATPase expression, reflecting improved calcium homeostasis and contractability over a four-week oral dosing regimen post–myocardial infarction. Through histological analysis, the authors reported reduced collagen deposition and decreased tumour necrosis factor alpha, highlighting the LASSBio-294 anti-fibrotic and anti-inflammatory properties. The effects were concentration dependent, with higher levels producing more profound benefits, emphasising the therapeutic potential of LASSBio-294 for managing heart failure related to IHD [177]. Overall, the strengths of this study included the robust experimental design, featuring well-defined control groups and a diverse range of evaluation methods. Echocardiographic assessments permitted precise functional analysis, while histological examinations provided insights into both structural and inflammatory changes post-MI. The four-week treatment duration used in this study was sufficient to capture acute and subacute events, and the use of spontaneously hypertensive rats as a model adds further translational relevance given their similarities to human hypertension and heart failure conditions. These factors enhance the reliability and applicability of the findings towards potential clinical studies. However, while the results of this study suggest A_2A_R activation can mitigate inflammation and fibrosis within the cardiac context, the precise molecular mechanism by which this occurs remains unclear. Future studies should explore the specific signalling pathways involved as well as the possible non-target effects of LASSBio-294 on other adenosine receptor subtypes, which may have an influence on the agent’s overall therapeutic profile. A further aspect to be considered for clinical translation is the assessment of systemic effects, such as potential impacts on liver or renal function. Given the involvement of ARs in various physiological processes throughout the body, evaluating these effects is critical for a holistic understanding of the safety and efficacy of LASSBio-294. Undoubtedly, the study by da Silva et al. [177] provided strong evidence supporting the cardioprotective effects of LASSBio-294 in a hypertensive rat model of MI, highlighting its potential as a novel therapeutic agent in heart failure and, potentially, in IHD therapy. Through reducing inflammation, fibrosis, and dysfunction, LASSBio-294 alleviates key pathological processes in post-MI remodelling. In this regard, a recent investigation by de Souza Rocha et al. [209] has focused on adopting LASSBio-294 as the lead compound to develop a new series of methylated *N*-acylhydrazones as AR agonists, based on the earlier positive outcomes obtained in the rat model of MI [177,208,210]. From this molecular optimization study, new effective drug candidates were identified, such as LASSBio-1359 and LASSBio-1386 [211,212], confirming earlier findings indicating that the presence of a small lipophilic group (i.e., methyl, in the case of these analogues) on the acylhydrazone function can lead to improved cardiovascular effects [213,214]. Taken together, the extensive studies conducted on the LASSBio series suggest that addressing gaps, such as optimizing the structure of the agents (to enhance both the activity and AR isoform selectivity profiles), along with exploring the systemic effects, accurate mechanism of action and monitoring long-term outcomes, may help pave the way for the clinical development of LASSBio-294.

### 8.6. CPA (A_1_R Agonist)

The investigation reported by Joosen and colleagues [178] relates to significant findings regarding the therapeutic efficacy of the A_1_R agonist CPA (Ki ≈ sub- to low-nM on A_1_R, while the potency at other adenosine receptors is significantly lower–i.e., Ki ≈ 1000-fold less on A_2A_R, very low binding on A_2B_R, and only negligible agonist effects on A_3_R. Interestingly, on this latter subtype, nM potency is recorded for CPA as an antagonist [215,216,217,218,219]. This was tested in experiments focusing on preventing cholinergic symptoms and mortality in rats resulting from sarin poisoning, which is well-known to be linked to chronic cardiomyopathy and other cardiac pathologies [220]. Specifically, Joosen’s results indicated that higher CPA doses (0.5 and 2.0 mg/kg) completely prevented mortality and the onset of cholinergic symptoms, whilst lower doses (0.1 and 0.05 mg/kg) reduced symptom severity but were less effective in preventing death. These findings highlight the dose-dependent nature of CPA’s therapeutic effects and highlight its potential utility in the management of acute sarin poisoning. The study importantly identified a critical role for CPA-induced bradycardia and hypotension in limiting sarin distribution, although it focused largely on peripheral mechanisms without fully exploring the potential central nervous system or cardiovascular effects of CPA [178]. Nonetheless, the ability of CPA (and following analogues, e.g., 2-chloro-N^6^-cyclopentyladenosine, CCPA [35,221,222]) to induce this effect may prove pivotal in the context of identifying new therapeutics for IHD. The short duration of the study failed to highlight whether CPA confers cardioprotective benefits over time or induces adverse effects over time, which is critical for evaluating its clinical utility. Several studies have investigated the use of CPA in alternative conditions (both cardio-related and not), for instance, neuroprotection, anticonvulsant effects, glaucoma, hypertension, and microvascular dysfunction [218,223,224,225,226,227]. However, many of these failed to measure the cardiovascular efficacy of this compound. Overall, a greater understanding of CPA’s cardiovascular effects is desirable to explore therapeutic use in IHD, as the initial findings are promising.

### 8.7. PSB-15826 (A_2A_R Agonist)

PSB-15826 emerged as the most potent AR agonist (sub-µM EC_50_ on A_2A_R, in functional assays [179]) among a series of tested analogues (originally designed and developed within a research programme focusing on anti-inflammatory agents for inflammatory bowel disease[228]) in a study conducted by Fuentes et al. [179]. The investigations revealed that this agent effectively inhibited platelet aggregation induced by ADP, with an EC_50_ value of 0.32 ± 0.05 μmol/L, significantly outperforming both CGS21680 (i.e., an established adenosine receptor ligand widely used as a reference standard in pharmacological studies) [229] and adenosine itself [179]. PSB-15826 additionally demonstrated a remarkable ability to increase intraplatelet cAMP levels (EC_50_ of 0.24 ± 0.01 μmol/L) and inhibit P-selectin localisation (EC_50_ of 0.062 ± 0.2 μmol/L), indicating its multifaceted role in modulating platelet function. Its potency is attributed to favourable interactions with the A_2A_R [230], including pi-stacking interactions with the residue His264 (not conserved across the other AR subtypes), which may enhance the compound’s residence time and efficacy in receptor activation. This confirms that PSB-15826 is a potent A_2A_R agonist with pronounced antiplatelet activity. It represents a promising candidate for further development, potentially also towards new treatments for IHD and other cardiocirculatory diseases, considering the well-established involvement of blood platelets in playing a critical role in the pathogenesis of a variety of inflammation-based conditions affecting the cardiovascular system [29,231,232]. However, its potential systemic effects (e.g., blood pressure lowering) need to be further investigated for potential use in coronary heart disease, myocardial infarction, thrombosis, and stroke. Moreover, the study by Fuentes and colleagues [179] is a pre-clinical investigation conducted in isolated platelets (from young and healthy volunteers), thus performed in a setting that does not fully account for the complexities of systemic circulation, where multiple variables may alter drug efficacy and safety. To expand the translational applicability of the results, long-term studies assessing the safety and therapeutic effectiveness of PSB-15826 in a clinical context are also required to substantiate the therapeutic potential, along with evaluating the antiplatelet effects in older populations or those presenting with underlying health conditions, such as cardiovascular diseases, who would benefit most from antiplatelet therapies. Overall, the biomedical data on PSB-15826 highlight the significance of the A_2A_R in platelet activation, as well as the suitability of investigating AR synthetic ligands as potential therapeutic agents. PSB-15826’s ability to achieve superior efficacy and potency in comparison to earlier analogues (which were adopted as lead candidates in extensive drug discovery programmes) [228,233,234,235] demonstrates the potential for rational drug design in developing more effective molecules addressing the limitations of current drugs and pharmacological agents.

## 9. Insights into the Use of AR Synthetic Ligands in IHD

The clinical potential of AR synthetic ligands in the treatment of IHD is a subject of considerable interest, given the multifaceted role of adenosine in cardiovascular physiology and pathology [30,34,80,119,120,121,122,123,124,125,126,236,237]. ARs are distributed throughout the cardiovascular system and play critical roles in mediating various physiological responses, including slowing heart rate, vasodilation, cardio-protection, and modulation of inflammatory processes [119,122,123,124,237]. The therapeutic strategy of targeting these receptors with more specific synthetic ligands aims to exploit their protective effects during adverse events. However, the inherent complexity of adenosine signalling pathways represents a significant challenge. In this regard, the differential expression and functional roles of ARs in various cell types can lead to unpredictable outcomes when modulating these pathways [115,120,122,123]. Moreover, the possibility of determining receptor desensitisation and tolerance may complicate the long-term use of AR agonists, as chronic activation may diminish their therapeutic effects [103,238].

As research into adenosine signalling pathway deepens, the potential for innovative therapeutic applications of AR synthetic ligands becomes increasingly apparent; however, the therapeutic utility may be masked by the lack of selectivity towards AR isoforms in the first instance, as well as other GPCRs. Indeed, other classes of GPCRs may share significant structural homology with adenosine receptors (mainly within the rhodopsin-like class A GPCRs), potentially causing a ligand to activate unintended targets and produce off-target effects, as for instance may be particularly the case with other members of the so-called “purinergic receptors” family (e.g., P2X and P2Y receptors) [74,143,165,239]. Moreover, various other GPCR sub-classes are expressed in the cardiovascular system [240,241,242,243,244] and cells of the systemic circulation (e.g., platelets) [245,246,247,248], thereby mediating physiological and pathological effects in various contexts. Therefore, the technical difficulty in generating specific GPCR class- and subtype-specific biological agents also needs to be regarded as a need, in order to access effective and safe drugs [249,250,251]. This is linked to another significant challenge, which relates to the side effect profile associated with AR ligands, as well as the receptor modulation itself [165]. In this regard, allosteric modulation represents a promising strategy in AR synthetic ligand therapy refinement. Positive AR allosteric modulators, which enhance the action of endogenous adenosine by binding to alternative sites on the receptor [68,238], have been proposed as a means to enhance therapeutic effects with reduced side effects. While this approach may offer greater physiological modulation of receptor activity, potentially reducing the likelihood of receptor desensitisation, as well as side effects associated with chronic activation [252], their clinical benefits remain to be fully confirmed at the present time. All these aspects need to be addressed to optimise the efficacy and safety of newly developed drug agents. As is often the case in the search for new medicines, the balance between achieving therapeutic goals and minimising adverse effects is delicate and necessitates careful consideration during drug development.

The pharmacokinetics of adenosine (and its synthetic ligands) further complicate the clinical application [25,115,117,172,176]. Adenosine is quickly metabolized in vivo, leading to short half-lives that necessitate frequent dosing or continuous infusion for effective therapeutic levels. This rapid clearance can result in fluctuating plasma concentrations, which may hinder the achievement of stable therapeutic effects. Additionally, the presence of endogenous or newly generated adenosine (in a pathological setting) can create a competitive environment that diminishes the efficacy of synthetic agents, particularly in pathological states where adenosine levels are elevated, such as during ischemia [24,93,96]. Addressing the pharmacokinetic challenges of adenosine and AR synthetic ligands is vital to advance their clinical use. Emerging approaches such as nanoparticle-based delivery systems enable the controlled release and targeted AR synthetic ligand delivery, improving bioavailability and metabolic stability [253]. These systems prolong the half-life of the drug agent in addition to reducing systemic exposure, thereby enhancing therapeutic efficacy whilst minimising adverse effects. In parallel, pro-drug development is also being explored to overcome the AR synthetic ligand current rapid clearance and achieve sustained receptor engagement [254]. Additionally, the use of combination therapies that target multiple pathways involved in diseases, including IHD [9], may enhance the overall therapeutic effect.

Overall, the development of novel AR synthetic ligands has advanced significantly in recent years [134,157,165,177,228,235], also through the introduction of innovative drug discovery approaches, such as structure-based drug design, virtual screening, and other computational modelling techniques, which take advantage of the artificial intelligence progression in the current epoch. These in silico screening tools can drastically enhance the identification of novel ligands with improved receptor selectivity and pharmacological profiles, enabling predictive interactions with their respective binding sites and optimizing efficacy towards the particular biological target of interest. Additionally, computational methods can also help to reduce off-target effects, thereby accelerating the process of translation from bench-lab discovery to clinical application. Lastly, it is also worth considering that the heterogeneity of IHD itself presents a challenge for the development of adenosine-based therapies. IHD encompasses a spectrum of conditions characterised by varying degrees of ischemia, myocardial injury, and patient-specific factors such as comorbidities and genetic predispositions [8,255]. These variabilities complicate the identification of suitable patient populations that would benefit most from AR modulation. Presumably, the integration of personalised medicine approaches, which consider individual patient characteristics, may be highly necessary in this context to optimise therapeutic outcomes.

## 10. Conclusions and Outlook

In conclusion, while AR synthetic ligands hold promise for the treatment of IHD, several challenges must be addressed to realise their full potential. The complexity of adenosine signalling, pharmacokinetic limitations, side effect profiles of potential therapeutic agents, as well as the heterogeneity of IHD, all contribute to the difficulties encountered in developing effective drugs and therapies. Future research should focus on innovative strategies to overcome these hurdles, including the exploration of novel ligands, combination therapies, and personalised medicine approaches. In this regard, imminent research priorities would definitely include the development of ligands with AR subtype selectivity and/or tuneable biased effects (to limit the triggering of adverse effects), as well as formulations and/or drug release systems to optimize timing and delivery strategies (e.g., ensuring drug exposure during the initial phases of reperfusion). A primary focus of further investigations should be cantered on improving pharmacokinetics (e.g., to address ultrashort half-lives, rapid systemic clearance, inefficient membrane permeability and off-target effects.) and myocardial targeting profile of new drug agents. In parallel, human-like in vivo models (along with clinically relevant comorbidities) should be adopted preclinically to assess the translation efficacy of potential new drug candidates, along with combination therapy approaches, to explore the suitability of multimodal or synergistic effects. Lastly, during clinical trials, accurate patient selection, correct administration timing, and route should be optimized, as well as the whole regimen, particularly in the case of chronic use. By addressing these challenges, the therapeutic application of adenosine synthetic ligands may ultimately lead to improved IHD therapies.

As researchers continue to investigate the adenosine signalling pathway and its implications for cardiac health, the landscape of AR synthetic ligands is likely to evolve. Challenges faced by current drug candidates should not dissuade further exploration but rather prompt a refined understanding of their mechanisms and functions. By bridging the gap between pre-clinical findings and clinical applicability, the potential of discovering AR-based therapies for IHD could constitute an exciting frontier in cardiovascular medicine.

## Figures and Tables

**Table 1:** Summary of adenosine effects, via distinct AR isoforms, on the cardiocirculatory system. Upward arrows = increase of activity; downward arrows = decrease of activity.

Effect	ARs	Mechanism	Outcome
Bradycardia	A_1_	Gi → ↓ cAMP → ↑ K^+^ efflux	Persistently slows heart rate
Atrioventricular node block	A_1_	Gi → ↓ cAMP → ↑ K^+^ efflux	Interrupts reentrant tachycardia
Coronary vasodilation	A_2A_, A_2B_	Gs → ↑ cAMP in smooth muscle NO ↑, ↑ K+ efflux	↑ Coronary flow
Hypotension	A_2A_	Systemic vasodilation	↓ BP
Anti-platelet	A_2A_	Gs → ↑ cAMP in platelets	↓ Aggregation
Ischemic protection	A_1_, A_2A_, A_3_	Multiple	↓ Injury during MI

**Table 2:** Summary of adenosine effects, via distinct AR isoforms, on the immune system. Upward arrows = increase of activity; downward arrows = decrease of activity.

Immune Cell Type	ARs	Effect
T cells	A_2A_, A_2B_	↓ T cell activation, ↓ IL-2 production, ↓ proliferation
Macrophages	A_2A_, A_2B_	↓ Pro-inflammatory cytokines (e.g., TNF-α, IL-6), ↑ IL-10
Neutrophils	A_2A_	↓ Chemotaxis, ↓ degranulation, ↓ reactive oxygen species (ROS)
Dendritic cells	A_2A_	↓ Maturation and antigen presentation
Mast cells	A_2B_, A_3_	↑ Histamine release (context-dependent), ↑ pro-inflammatory cytokines
Natural Killer (NK) cells	A_2A_	↓ Cytotoxicity and cytokine secretion

**Table 3:** Chemical structures of AR synthetic small-molecule ligands, along with subtype receptor target, compound name, and objective of the clinical evaluation.

AR Ligand	Structure	AR	Objective	Potency
**RPR-749** [172] (clinical stage)	*structure not available (Aventis, 1429209-67-5)*	A_1_	lipid concentration activity	low-nM Ki
**LJ-1888** [173,174] (9d in [180]) (pre-clinical stage)	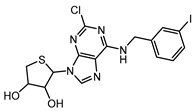	A_3_	cardioprotection	Ki ≈ 10–30 nM
**IB-MECA** [175] (pre-clinical stage)	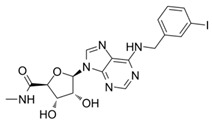	A_3_	cardioprotection	Ki ≈ 1.1 nM
**GS-9667** [176] (clinical stage)	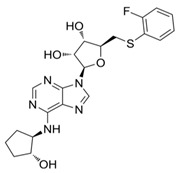	A_1_	lipid concentration activity	Ki ≈ 55 nM
**LassBio-294** [177] (pre-clinical stage)	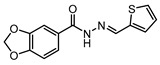	A_2A_	cardioprotection	µM EC_50_
**CPA** [178] (clinical stage)	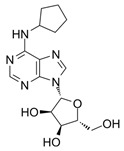	A_1_	cardiovascular effects	sub- to low-nM Ki
**PSB-15826** [179] (clinical stage)	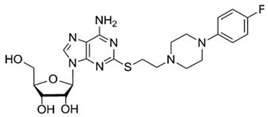	A_2A_	antiplatelet activity	sub-µM EC_50_

## Data Availability

Not applicable.
